# A lottery incentive system to facilitate dialogue and social support for workplace HIV counselling and testing: A qualitative inquiry

**DOI:** 10.1080/17290376.2014.937739

**Published:** 2014-07-15

**Authors:** Martin Weihs, Anna Meyer-Weitz

**Affiliations:** ^a^ MBA, MPhil, is affiliated as a technical advisor to the Automotive Industry Development Centre Eastern Cape (AIDC EC) placed by the German Development Cooperation (GIZ), Port Elizabeth, South Africa; ^b^ PhD, is affiliated as Professor to the School of Applied Human Sciences, Discipline of Psychology at the University of KwaZulu-Natal, Durban, South Africa

**Keywords:** HCT, company, collectivist, group pressure, norm, South Africa, CDV, entreprise, collectiviste, pression de groupe, norme, Afrique du Sud

## Abstract

Despite South African mid-sized companies' efforts to offer HIV counselling and testing (HCT) in the workplace, companies report relatively poor uptake rates. An urgent need for a range of different interventions aimed at increasing participation in workplace HCT has been identified. The aim of this study was to explore qualitatively the influence of a lottery incentive system (LIS) as an intervention to influence shop-floor workers' workplace HIV testing behaviour. A qualitative study was conducted among 17 shop-floor workers via convenience sampling in two mid-sized South African automotive manufacturing companies in which an LIS for HCT was implemented. The in-depth interviews employed a semi-structured interview schedule and thematic analysis was used to analyse the data. The interviews revealed that the LIS created excitement in the companies and renewed employees' personal interest in HCT. The excitement facilitated social interactions that resulted in a strong group cohesion pertaining to HCT that mitigated the burden of HIV stigma in the workplace. Open discussions allowed for the development of supportive social group pressure to seek HCT as a collective in anticipation of a reward. Lotteries were perceived as a supportive and innovative company approach to workplace HCT. The study identified important aspects for consideration by companies when using an LIS to enhance workplace HIV testing. The significance of inter- and intra-player dialogue in activating supportive social norms for HIV testing in collectivist African contexts was highlighted.

## Résumé

Malgré les efforts que font les petites et moyennes entreprises sud-africaines pour offrir le conseil et dépistage volontaire du VIH (CDV) en milieu de travail, les entreprises font état de taux de participation relativement bas. Un besoin urgent pour une gamme de différentes interventions visant à accroître la participation au CDV en milieu de travail a été identifiée. Le but de cette étude était d'étudier qualitativement l'influence d'un système d'incitation à la loterie comme intervention pour influencer le comportement CDV des ouvriers en milieu de travail. Une étude qualitative a été menée auprès de 17 ouvriers choisis par échantillonnage de commodité dans 2 entreprises de fabrication automobile en Afrique du Sud. Dans ces 2 entreprises de taille moyenne, un système d'incitation à la loterie a été mis en œuvre pour encourager le CDV en milieu de travail. Un guide d'interviews semi-structurées a été employé pour mener des entretiens approfondis. L'analyse thématique a été utilisée pour analyser les données. Les entrevues ont révélé que les systèmes d'incitation ont créé de l'excitation dans les entreprises et ont renouvelé l'intérêt personnel des employés pour le CDV. L'excitation a facilité des interactions sociales qui ont abouti à une forte cohésion du groupe d'ouvriers concernant le CDV et atténuant ainsi la stigmatisation liée au VIH en milieu de travail. Des discussions ouvertes ont permis l'élaboration de pression sociale de groupe en support à la participation collective au CDV en prévision d'une récompense. Les loteries ont été perçues comme une approche soutenante et innovante de la part des entreprises pour le CDV en milieu de travail. L'étude a identifié des aspects importants à prendre en compte par les entreprises lors de l'utilisation d'un système d'incitation à la loterie pour augmenter le taux de dépistage VIH en milieu de travail. L'importance du dialogue inter- et intra-joueur dans l'activation de normes sociales favorables pour le dépistage VIH dans les contextes collectivistes africains a été soulignée.

## 1. Introduction

### 1.1. The urgent need for increasing participation in workplace HIV counselling and testing

It is estimated that only half of the approximately 34 million people living with HIV at the end of 2011 knew their HIV status (UNAIDS 2012). As globally 90% of the people living with HIV are in the most productive period of their lives, be they workers, managers or employers (SABCOHA 2012), health management of employees is becoming a common imperative for companies that do business in regions where there is an HIV epidemic (George & Quinlan 2009). No actual estimates exist about the HIV prevalence within the South African workforce, but the HSRC estimated that 6.4 million people were living with HIV in 2012 in South Africa and that only 44.8% were aware of their HIV status (Shisana 2013). In response to the magnitude of this epidemic, the South African National Strategic Plan on HIV, STIs and TB, 2012–2016 foresees that all workplace wellness programmes should address HIV, STIs and TB in an integrated manner and aligned with national standards, specifically the South African HIV National Standard for Workplace Programmes, SANS 16001 (DOH 2011). Private sector HIV and AIDS workplace programmes (WPPs) in South Africa have evolved considerably (GBC and IFC 2010) and by motivating employees to seek HIV counselling and testing (HCT) in the workplace, businesses support the National Strategic Plan priority in ensuring that 80% of adults in South Africa will know their HIV status by 2016 (DOH 2011).

The workplace has been found to be the ideal environment for tackling the epidemic as employers and employees group together regularly in an environment where an ‘organisational culture’ offers a consistent platform from which a comprehensive HIV and AIDS WPP can be implemented (SABCOHA 2012; Van Dyk 2008). Hence, the workplace is regarded as a good setting to reach men and women of reproductive age when it comes to HIV prevention, including HCT, and care (Bhagwanjee, Petersen, Akintola & George 2008; Mundy & Dickinson 2004; SABCOHA 2012; Van Dyk 2008). Moreover workplace HCT contributes to effective health management in the early identification of HIV and timely management of HIV infection in the pre-symptomatic stage of AIDS, leading to lower operational costs for both the HIV and AIDS WPPs and business production (George & Quinlan 2009). For some companies such as Debswana, voluntary workplace HIV testing served as a turning point in efforts to address HIV and AIDS as the anonymous statistics about HIV testing allowed for detailed understanding of the extent to which the company was affected by HIV and AIDS (UNAIDS 2002). HCT is also considered to be one of the most effective and economically viable strategies for HIV prevention and access to care in Africa (Corbett, Dauya, Matambo, Cheung, Makamure, Bassett, *et al*. 2006). Moreover it has been argued that HCT constitutes a valuable goal for South African companies, as knowing their HIV status is more likely to ensure health protection for employees and for their partners, families and communities (Coates, Richter & Caceras 2008; Centers for Diseases Control and Prevention 2013; Van Dyk 2008). However, it should also be noted that employees' workplace HCT behaviours are complex and may be influenced by various factors.

In their 2008 study, Bhagwanjee *et al.* attributed the success of a workplace HCT campaign in a South African mining company to the convenience of its on-site nature and the use of rapid testing, thereby making results easily available. While the campaign's pre-test counselling information was also reported as being important in alerting employees about the benefits of HIV testing, the group nature of the HCT campaign seemed also to have encouraged many employees to test thanks to increased peer influences. Then too the participation of senior management in HCT campaigns, where short and intensive know-your-status initiatives were offered, significantly increased workplace HCT participation (SABCOHA 2012).

Yet, despite South African companies' efforts to offer HCT in the workplace, companies report HCT uptake rates that are seldom higher than 45%, due to stigma, denial, fear and ignorance (SABCOHA 2012). Similarly in earlier studies, Connelly and Rosen (2006), George (2006) and George and Quinlan (2009) found that many South African companies reported relatively poor HCT uptake rates. It appears that HCT uptake in the workplace is hampered by inter alia perceptions about a lack of confidentiality and fear of stigmatisation and discrimination in both the workplace and community in the event of testing HIV positive (Bhagwanjee *et al*. 2008; Young & Bendavid 2010). Therefore employees might decide not to participate in workplace HCT so as to avoid the stigmatisation associated both with HIV testing and a likely positive HIV test result.

Furthermore, evidence supports the possibility that some people seek cover for HIV testing (Young & Bendavid 2010). For example, encouraging people to donate blood may provide a cover for HIV testing as it allows people to appear interested in performing the non-stigmatised service of blood donation (Chiavetta, Ennis, Gula, Baker & Chambers 2000; Korelitz, Busch & Williams 1996). Similarly, a study conducted in a large South African company by Arimoto, Ito, Kudo and Tsukada (2013) indicated that providing workers with an excuse to participate jointly in workplace HCT might be more effective than just offering voluntary and confidential workplace HCT to individuals. Not surprisingly therefore, Bhagwanjee *et al.* (2008), Arimoto *et al.* (2013), and SABCOHA (2012) all point to an urgent need for a range of different interventions aimed at increasing participation in workplace HCT.

### 1.2. Lottery incentive systems to encourage HCT

In the review on the effects of economic incentives on consumers' health-preventive behaviours, economic incentives are described as a wide variety of actions, including cash payments, lotteries, coupons for free or reduced-price goods and services, gifts, free or reduced-price medical services, and the opportunity to avoid disincentives (Kane, Johnson, Town & Butler 2004). Whilst cash incentives as conditional cash transfers allow all recipients to receive cash if they fulfil various requirements, such as a desired HIV-preventive behaviour, in a conditional lottery all those who participate in a desired HIV-preventive behaviour, such as HIV testing, are entered in a lottery but only a few have a chance to win a prize. There is a body of literature which covers cash incentives and HIV behaviour change (Kohler & Thornton 2011; Operario, Kuo, Sosa-Rub & Galárraga 2013; Pettifor, MacPhail, Nguyen & Rosenburg 2012; de Walque, Dow, Nathan, Abdul, Abilahi & Gong, *et al.* 2012) but very little exists on the use of lotteries for HIV behaviour change.

The use of lottery incentives for health goes back to the well-cited 1957 Glasgow story in Scotland, where incentives were used to motivate for early TB screening in an attempt to address the high TB death rate in Britain at the time. The aim was to get 250,000 people over a five-week period to seek lung X-rays but instead about three times more people tested in one week for the weekly price draw (The Glasgow Story 2014). In later years, cash lottery prices were used to motivate for the participation in vaccinations (Yokley & Glenwick 1984) and seek screening for cholesterol levels (Francisco, Paine, Fawcett, Johnston & Banks 1994). Lottery incentives are viewed to have significantly improved patient adherence to the blood-thinning medication Warfarin, which has potentially severe side effects (Volpp, Loewenstein, Troxel, Doshi, Price, Laskin, *et al*. 2008b) and to help Americans lose weight (Volpp, John, Troxel, Norton, Fassbender & Loewenstein 2008a).

The South African ‘Right to Know’ HIV testing competition (Keeton 2009) and the Western Cape Government (Mbhele 2011) have used lottery incentive systems (LISs) to encourage South African citizens to get tested for HIV. Similarly, some large South African companies of the automotive sector make use of LIS for workplace HCT as they believe that lottery incentives positively influence workplace HCT behaviour. The Chief Executive, Sean Jelly, of Lifeworks, a disease management company in South Africa, was cited by George and Quinlan (2009) to have noted that raffling a weekend family holiday amongst those who attend a VCT exercise greatly increased workplace HIV testing uptake. However, few empirical studies exist on why and how LIS schemes influence employees' workplace HCT behaviour in general and particularly in the South African automotive supplier sector. According to Lee, Cui, Muessig, Thirumurthy and Tucker (2013) more research on the theoretical underpinning of incentives used for testing is necessary. For this reason the current qualitative study aims to provide a better understanding of employeeś experiences of a workplace LIS to encourage workplace HCT in two independent mid-size automotive supplier companies of the Nelson Mandela Bay, South Africa.

## 2. Methodology

### 2.1. Research settings and study design

This qualitative study forms part of a quasi-experimental study using a sequential explanatory approach (Hanson, Cresswell, Plano Clark, Petska & Cresswell 2005) to assess the influence of an LIS in an HCT campaign conducted in two independent mid-sized automotive supplier companies of the Nelson Mandela Bay in South Africa. In this approach, the quantitative study (Weihs & Meyer-Weitz 2013) had the dominant focus while the qualitative study was employed to gain a deeper understanding of employees' experiences of the LIS as part of the HCT campaign. It is important to note that both these companies had never before made use of incentives to motivate their shop-floor workers to utilise workplace HCT and that both companies had offered free and voluntary workplace HIV testing in the past, but testing uptake rates did not exceed 30% of staff.

Prior to workplace HCT, the targeted workers in both companies benefited from on-going comprehensive HIV and AIDS WPPs for at least a six-month period. The WPP curriculum focussed on HIV and AIDS company policy issues, HIV and AIDS education and skills training for HIV prevention, condom use and HCT negotiations.

The first step of the experimental intervention was the announcement of the LIS. A leaflet was distributed to all the shop-floor workers approximately two weeks before workplace HIV testing, announcing that participants in workplace HCT would receive free t-shirts and would be entered into a company lottery which afforded opportunities to win gift cards (a first prize of 2000 ZAR, a second prize of 500 ZAR, and 10 extra 100 ZAR prizes). The first price was calculated to be close to half a month's shop-floor worker's wage (average monthly salary of a shop-floor worker in both companies approximated 5000 ZAR). Peer educators explained the leaflet content to the workers, clarifying that testing for HIV was a precondition for entering in the lottery, that the lottery draw would take place in the presence of all staff the day after HIV testing and that the winners would receive their gift vouchers from managers immediately after the draw. Posters that announced the LIS were also displayed in both company workplaces. The lottery draws took place shortly after the workplace HCT and the winners received their gift vouchers in the presence of all staff.

### 2.2. Sampling and data collection

For greater credibility of the findings, site triangulation was achieved by the participation of informants within two organisations so as to reduce the effect on the study of particular local factors peculiar to one institution (Shenton 2004). The sample comprised 17 participants (9 and 8 from the respective companies) from the experimental study population (*N* = 203) exposed to the LIS, irrespective of whether they sought HIV testing or not. Due to logistical reasons, the study participants were conveniently selected by the various shift leaders according to availability. The principle of data saturation as explained by Fossey, Harvey, McDermott and Davidson (2002) was used in determining the final sample size (*N* = 17) to prevent unnecessary repetition and also to account for possible selection bias. Due to the explanatory nature of the study, the sample size was considered adequate. In Table 1, the characteristics of the sample are presented. The majority of the employees was younger than 30 years and had obtained a Grade 12 level of education. There were slightly more females than males and about an equal number of married and single participants. While not selected on the basis of their uptake of HCT, all the participants indicated that they tested for HIV and were thus entered into the lottery.

**Table 1.** Characteristics of shop-floor workers (*N* = 17).

Characteristics	Number
*Age in years*	
20–29	7
30–39	6
40–49	3
50–59	1
*Sex*	
Female	9
Male	8
*Employment status*	
Contractor	6
Permanent	11
*Marital status*	
Single	9
Married	7
Divorced	1
*Highest education reached*	
High school	6
Matric	9
College	2
*Tested in workplace HCT*	
Yes	17
No	0

The interviews for this study were conducted in December 2010, about two weeks after the workplace HIV testing initiative and the lottery draw had taken place in both companies. The HCT uptake rate in both companies was above 85% compared to an average below 30% prior to intervention. Two interviewers using a semi-structured interview schedule conducted the in-depth interviews in the companies' canteens. Although the canteens are shared by all employees, care was taken to ensure that the interviews could not be overheard by other people. The interview process could however be observed by colleagues and while it was expected that this might inhibit response, the researchers were pleasantly surprised by the active engagement of the employees. Participants were encouraged to share their personal and honest views as suggested by Shenton (2004). The interviewers indicated that there were no right or wrong responses and emphasised their independence from the companies. Before commencing the interviews, the aims and objectives of the study and voluntary nature of participation as well as issues of confidentiality and anonymity of the data and their right to withdraw from the study without justification were explained. Informed consent for participation and permission for the audio taping of the interviews were obtained. The interview guide covered a range of issues, for example, general experiences of the LIS, ways in which the LIS could have influenced HCT with consideration of lottery prizes, their own uptake of HCT and personal experiences of the LIS. All interviews were conducted in English as the candidates had sufficient English language competency to understand the questions and to answer in English. Care was taken during the interview process to paraphrase responses to ensure authenticity of the responses. Ethical approval for the study was obtained from the University of KwaZulu-Natal in October 2010 (reference number HSS/1263/010).

### 2.3. Data analysis

All interviews were transcribed verbatim for the purpose of data analysis. The computer software package Atlas.ti was used to manage and code the data. No specific research question informed the data analysis. Instead thematic analysis was conducted as outlined by Braun and Clarke (2006). The entire data set was systematically processed and interesting aspects were identified so as to form the basis for repeated themes across the data set. Direct quotations illustrated these identified themes and analysis was then conducted on a purely semantic level. Analysis was conducted within realistic/essentialist paradigms and motivations. Experiences and meanings were thus theorised in a straight-forward way because a largely unidirectional relationship was assumed between meaning, experience and language (Potter & Wetherell 1987).

## 3. Findings and discussion

The study generated a wide range of themes relating to workers' experiences of a lottery system as part of a workplace HCT campaign in the three weeks between the LIS announcement and the lottery draw. The main findings from the analysis of the qualitative data are organised into four sections, namely renewed personal interest in HCT, open HCT talk and soliciting of group support for HCT, adhering to group norms for workplace HCT behaviour and LIS as signal of company concern for employees.

### 3.1. Renewed personal interest in HCT

Despite companies' six months of comprehensive HIV and AIDS campaign activities, interviewees stated that many of their colleagues, the companies' cleaners and they themselves had been unwilling to participate in workplace HCT prior to the announcement of the lottery incentives. This is in line with research reporting that, even when companies tried to implement HIV and AIDS programmes, workplace HCT uptake was often low (Connelly & Rosen 2006; George 2006; George & Quinlan 2009; SABCOHA 2012). It also confirms the conclusion of Arimoto *et al.* (2013) that merely encouraging voluntary, private and confidential HIV testing in the workplace does not significantly increase HIV testing rates.

The focus on HIV and AIDS education both in the public domain and in the workplace might have desensitised many people for prevention messages including HIV testing (INTRAC 2008; SWHAP 2008). In addition, studies have found that talking about employees' HIV vulnerability in the workplace can be perceived as insulting. Furthermore, the workplace sometimes is looked upon as an escape from having to deal with the personal realities of HIV and AIDS issues amongst extended and immediate family at home (INTRAC 2008). Hence, the workplace is not necessarily an ideal and supportive environment for individuals to communicate with colleagues about HIV and AIDS. However, the lottery incentives were described as an innovative approach that helped change the employees' receptiveness for HCT messages that were previously not possible.

The study participants agreed that the LIS had increased the interest in workplace HCT, thereby encouraging shop-floor workers to consider participating in workplace HIV testing. The participants who stated that some employees had no interest in HIV testing prior to the LIS were of the opinion that the possibility of winning a prize played a role in motivating them for testing. As outlined above, it is a concern that despite all the educational interventions prior to the LIS, some employees did not show insight into the value of HCT as an important goal in itself, but that the likelihood of winning a prize seemed to be the sole motivating factor.

 … yes, especially the cleaners, they weren't interested in that [HCT]. But they also went.

At first there was like tests where you are giving your blood, you know, then you say ah ah, I'm not going to go because there is nothing for me, but now when you are introducing something, saying you will win something, a voucher or whatever, even if it was not a two thousand Rand voucher, you say, even a t-shirt, you feeling like, more, like more confident. Say, I'm going to do a blood test, here is something at the end of the day, so that is more.

 … before it was likely, I mean if there wasn't money for the testing, then the people would not have come for the testing, they will be, OK never mind, I will live with that, but the fact that there was two thousand Rand reward, so they had to go, it was good thing they do that, yes.

The motivating nature of rewards for behaviour as presented by an LIS has been supported by various other studies (Francisco *et al.* 1994; Volpp et al. 2008a, 2008b; Yokley & Glenwick 1984). People are motivated by the experience of past rewards and the prospect of future rewards (Kahneman & Tversky 1979; Loewenstein, Weber, Hsee & Welch 2001). A study by Haisley, Volpp, Pellathy and Loewenstein (n.d.) suggested that lottery approaches may be a particularly useful way to drive higher rates of engagement in health and wellness among lower income employees. At a cognitive level, Eccles and Wigfield (1995), Borders, Earleywine and Huey (2004) and Kane *et al.* (2004) found that when incentives are offered for a task, the expected value and costs of the task outcomes are assessed in the light of the size and nature of the incentives. The LIS seemed to have initiated an engagement with HIV testing but also a self-debate in which the costs and benefits of HCT were considered. The participants described how the LIS stimulated them to think about HCT.

For me, it was like, as I said I never went for test myself before, so I just thought that, if I could honestly say that, I thought to myself that the outcome of this was gonna be that there's only a few people that go for the test because many people said that were afraid to find out, if they have the virus. So I thought I stand a good chance for winning because it wouldn't be many people going, you know.

 … as I said this thing [the HIV testing competition] was announced way before it actually took place, so this kept me thinking so much, I was thinking about it over and over and over and over, so ya it did.

It was interesting to note how this self-debate focussed around fear of HIV testing in relation to possible stigma and discrimination as well as confidentiality issues of HCT within the workplace context.

Because as I said, most, most of us, most of us, were really afraid, you know, the stigma that is involved – because you hear what people have to say about it, you know, there're many myths about this thing and stuff like that – but because of these prizes, you know, euhmmm … like for me, I speak personally, I don't know for the other people, but really it did a big thing, … in going for the testing.

You are like willing to go for it, it's exciting also, it is the same as that one, because the minute you see there is something – your mind is saying, let me go for it because anyway I'm going to win something, so I can do it. I can do it, it is like that. It becomes easier, every day, every day, it is easier.

Ya, ya just trying to find out, Eeerm, to me, Eeerm, when I heard about the, the testing I said: Hey, why inside the company? Because I wanted to do it, maybe at the doctor or private place, but, hey, but the company! Eeerm, there was that prize, everyone is doing it, why can't I do it. Ya it helped.

The quotations above reflect how the participants weighed the costs and benefits of workplace HCT and the role of the lottery in this regard. The lottery event influenced their motivation to test and seemed to have assisted the participants to overcome their individual concerns about the likely outcome of the HIV tests. Not only did they rationalise their fears, but it was also very clear that they perceived other employees to have the same concerns, but nevertheless assumed that others would test and thus felt confident that they would be able, like others, to overcome their fears and seek HCT. Thornton (2005) argued that incentives may directly compensate for HIV- and HCT-related fears. The prospect of perhaps winning the lottery increased participants' confidence and willingness to undertake HIV testing.

Ok, everybody is scared, you know. But incentives brought a lot of people that didn't want to be tested … Lots that I know who feared this in the company … said that they are not going. But when they heard about the raffle then they came, you know. Because, everybody else needs money nowadays.

The South African literature talks about the fear of stigmatisation and social marginalisation if presumed to be HIV positive by attending workplace facilities as major workplace HCT barriers (Day, Miyamura, Grant, Leeuw, Munsamy, Baggaley, *et al.* 2003; Ginwalla, Grant, Day, Dlova, MacIntyre, Baggaley, *et al.* 2002; Kalichman & Simbayi 2003; Nuhawa, Kabatesi, Muganwa & Whalen 2002; Wolff, Nyanzi, Katongole, Ruberantwari & Whitworth 2005). Furthermore, believing that a positive test result leads to societal rejection is a critical barrier to HCT (Baggaley, Kelly, Weinrich & Kayawe 1998; Maman Mbwambo, Hogan, Kilonzo & Sweat 2001). Perceived employer, supervisory or co-worker hostility have been strongly associated with a failure to participate in testing for HIV (Mundy & Dickinson 2004). Furthermore, the perceived violations of confidentiality are critical barriers to HCT uptake (Day *et al.* 2003; Ginwalla *et al.* 2002; Kalichman & Simbayi 2003). Other barriers to HIV testing are reported by Bhagwanjee *et al.* (2008), i.e. uncertainties around the voluntary basis of HCT services and the group nature of HCT workplace initiatives as colleagues might detect test results due to the psychological reactions when testing positive. It appears that HCT uptake in the workplace is hampered by perceptions about a general lack of confidentiality and fear of stigmatisation and discrimination within both the workplace and community in the event of testing HIV positive (Bhagwanjee *et al.* 2008; Young & Bendavid 2010). However, the LIS seemed to have downplayed these obstacles normally encountered as it was argued that all employees had similar concerns but that all wanted to win.

To avoid the potential stigmatisation associated with HIV testing, individuals who wish to test for HIV may attempt strategies to avoid this stigma (Padilla, Castellanos, Guilamo-Ramos, Reyes, Marte & Soriano 2008; Young & Bendavid 2010). Interviewees described how they were able to conceal their interest in testing as the LIS gave them non-stigmatised reasons for participating in workplace HCT:

Because, everybody else needs money nowadays

 … because anyway I'm going to win something

Bulungula Incubator, the health co-ordinator of Nomzingisi Hopisi, reported in a newspaper interview that her project had experienced similar results using lottery prizes for HCT in a South African village: People can say: ‘Well I'm just going to try to win a cell phone’. They don't have to say: ‘Well, I am going because I think I may have HIV’ (Malan 2013). Interviewees had good excuses on individual and group levels to participate in workplace HCT and so the LIS helped mitigate the burden of possible stigma and discrimination. Hence, it is clear that providing workers with an excuse to participate in workplace HIV testing along with the confidential HCT procedure is likely to be effective when introducing workplace HCT (Arimoto *et al.* 2013).

Apart from cognitive engagement regarding HCT, the LIS impacted on employees' affect. An overwhelming number of interviewees described how LIS had been perceived by staff as an innovative approach to workplace HCT thereby creating positive emotions and excitement amongst shop-floor workers:

It (the LIS) made me eager to go. Because of the prices and because of the voucher. It actually made you feel good. Because it is like, you know, the people who is doing the testing and who, who is doing the testing, ne, they care for you. It is like they care for you, they even offer you something in return.

It made me more eager and happy I would say to go on testing … just being part of it ya, being part of it, I enjoyed myself

Ya, there was a huge drive; here in the company there was a huge drive. Everyone was excited, everyone was talking about it and there was huge encouragement. It was communicated in a way that everyone had to attend, most of our people went for the test.

 … for the incentives and even for the wellness period that we had, it was great, it was awesome, because we never had this in our company before. We only have a World AIDS day, but that was more about awareness for people at work and safety as well and health as well, but it was very awesome.

According to Airhihenbuwa and Obregon (2000) decisions about preventing HIV and AIDS are based on emotions and thus may not follow any of the pre-established patterns of decision-making advanced in many of the theories and models. As the LIS seemed to have enhanced both self-talk and positive emotions about HCT, it is likely that a greater readiness to test for HIV was created. The lotteries brought in a new and different approach to an old issue, allowing a new way to think and feel about HIV but also to talk about HCT in the workplace. The excitement around the lottery energised the group of shop-floor workers to discuss the prizes and HCT matters openly as described below.

### 3.2. Open HCT talk and soliciting of group support for HCT

Workplace HCT issues were taken from an individual level to a social level as workers interacted in the workplace with colleagues, for example, about the lottery incentives, about mutual encouragement to seek HCT to be entered in the lottery, and about their fears and experiences with testing for HIV.

Many participants reported how lottery incentives gave workers a company-wide accepted reason for starting to talk with colleagues about the prizes and workplace HCT, to share jokes and dream about winning and sharing the prizes:

Ya everyone was talking about it. The lucky draw and the testing.

Yes exactly, you will find like people who's going, you can even talk about it (workplace HCT), you can even ask, you can even like make some jokes, you know, about it, so it's like, it's more confident, like you are confident about this. It becomes easier, everyday, everyday, it is easier.

And I told my colleagues, we spoke to each other. When we speak and make jokes and I say … If I win or you win, then we going to share the money.

These findings were also supported in a South African community project that used lottery prizes to motivate for the uptake of HCT. The health coordinator of Nomziginisi Hopisi said: ‘Our raffles and goodie bags make people talk about HIV. And the more they talk about it, the more normal the condition becomes and the more willing they become to take steps, such as testing for HIV, to prolong their lives’ (Malan 2013).

The participants in our qualitative study described how group talks allowed them to clarify their focus on winning the two thousand Rand and for this reason they had to participate in HCT:

 … their mind-set was that they were going to win so they have to go for that.

Talks had also allowed group members to motivate each other to participate in workplace HCT. A participant described how the group goals were negotiated for all to participate in workplace HCT:

Most of the people participated and it was actually a goal for us 303 people.

And all the people were talking, ya. All my friends, my department, all of us went, talking you know, I had to go.

 … it was that they want to win that money, they want to win that prizes that gave me the encouragement, no they have to go for it … 

Ya because it motivates me as well to go, so if I tell them [work colleagues] look here there's an incentive and I say ‘Oh no!, I don't feel like going’, then they will also motivate you like ‘No but you must go, you can bring two thousand Rand home’ and they encourage you to go … .

As seen above, an overwhelming number of interviewees described how supportive the created group pressure was to seek HCT. Supportive group pressure, a characteristic of collectivist societies (Van Dyk & Kock 2004), was built up before the testing event to seek group participation in the forthcoming workplace HCT. Viewed from an individualistic perspective, it can be argued that employees decreased their chances to win the lottery by encouraging all their colleagues to test for HIV. These findings reinforce the views of Van Dyk (2001) who highlighted the significance of health-care interventions that are cognisant of collective norms in traditional African contexts.

It was however not only because of the likelihood of winning a prize that employees wanted to seek HCT. An interesting view was expressed that the talk about HIV testing created an interest to know one's personal HIV status.

And because everyone was talking about it, it made me like curious to know my status.

Some employees wanted to test for HIV irrespective of the LIS but as a goal in itself – to seek clarity about a possible positive HIV status.

But I told them, even if there wasn't that two thousand Rand – I still want to go, I still feel like I want to go because some of my friends told me they're not going. But I told them, what if you are maybe positive, it's better to find out.

The above views clearly show how group members played a role in encouraging each other to test in anticipation of a reward and through this process testing became a group activity rather than a lonely individual event. In addition, these talks supported the primary health message that it is important to know one's HIV status while also providing mutual support in this regard.

Moreover, the LIS enabled participants to communicate about HCT and LIS with family, friends and partners. Employees informed their family and friends at home about the HCT campaign.

I told my wife, listen there's this campaign coming, it's to do with HIV and AIDS, what happens is people, there's gonna be like this huge testing thing, whereby you can test for like diabetes, high blood pressure all that type of stuff and HIV test … 

Participants also gained support for participating in the workplace HCT by discussing the LIS with their families and partners.

But the day when you told us that there is going to be prizes, then I went back to them and said: Hey there are more prizes you know, even that, that final day of the testing there is a raffle also included and … someone will win, so communicating between me and my family … that also encouraged me to go to do those tests, it was like that.

It seemed that female partners were sensitive to their male partners' hesitation about testing and provided some assurance and even encouragement to test as seen below:

 … I spoke to her [partner] and she was like, don't worry baby, you gonna win that two thousand Rand voucher for us, you know.

She did very much encourage me, she said go for it, do it.

When the opportunity for company HIV testing came, workers volunteered as a group and members supported their hesitant colleagues.

### 3.3. Adhering to group norms for workplace HCT behaviour

The HCT campaign in the context of the LIS was transformed into a group ‘project’ where mutual encouragement and strong peer pressure to test played a role in the uptake of HCT. Peer pressure can be used to good effect in HIV programmes (Ghielen n.d.). A peer educator described how the group argument for a chance to win the lottery prizes was repeated amongst the group members to motivate each other to participate in the HIV test:

And I took everybody with me and I said, come, you all have the chance to win the two thousand Rand, so … that's how it influenced me.

The group seemed to have understood the hesitation and possible fear surrounding HIV testing but the idea of a collective action made it easier for people to test.

Because if one person sees, boy there's lots of people going, they gonna go.

These findings illustrate how a high priority was given to in-group interests which have precedence in collectivist societies. Because actions are guided by consideration of the in-group interests in these societies, group goals will take priority over individual goals (Jackson, Colquitt, Wesson & Zapata-Phelan 2006). The behaviour of the individual in relation to family and community is a major cultural factor that has implications for sexual behaviour and HIV and AIDS prevention (Airhihenbuwa & DeWitt Webster 2004). The above-described priority that was given to in-group interests together with the influence coming from family members suggest that employees' HCT behaviours were influenced by colleagues and family. This supports Airhihenbuwa and DeWitt Webster's (2004) finding that in the African context the values of extended family and community significantly influence the behaviour of the individual. It is therefore likely that HCT behaviour may become sustainable beyond the life of the intervention exactly because of collective consensus to test for HIV as argued by Airhihenbuwa and DeWitt Webster (2004).

When observing an excited majority of people going to test for HIV while smiling, and their willingness to openly discuss testing, hesitant individuals were encouraged to participate spontaneously in workplace HCT:

No, there was no one who exactly was pushing me. But, at first when I heard about this programme, no I was just OK I will see, but I wouldn't go for testing, but as I see the majority of people go, coming in there, testing, coming out, excited, wonderful, they discuss it and all that stuff. I was like, hey, it's like I was the only one who didn't go for testing but, I wasn't planning to go to do testing this year. I was planning to do may be … like next year … but I, I felt pressurized … They didn't say … but I did go for testing.

This excerpt shows how perceived descriptive norms, which are based on observation of what people typically do (Cialdini 2003), positively influenced participants' HCT behaviour. Findings also indicate that injunctive norms positively influenced HIV testing behaviour as colleagues' approval of HIV testing (shown with smiles and excitement when going in and out of HCT venues) strongly motivated hesitant participants to join the group. As pointed out by Cialdini (2003) when descriptive norms are aligned with injunctive norms, the power of normative appeals is optimised.

A major outcome of the communication process seemed to be the generation of group-based meaning- and decision-making around HCT and lottery incentives. The dialogues among shop-floor workers facilitated new collective meanings and sense-making about HIV in the workplace, thus resulting in shifts from previous negative perceptions of HIV testing in the workplace to more supportive views. A process of shared ‘sense making’ through inter-individual communication fostered the development of particular social representations about a particular aspect (Moscovici 1988) and, in this case, about testing for HIV within a workplace lottery system.

### 3.4. LIS as signal of company concern for employees

Production priorities hampering companies' HIV and AIDS WPP activities was a complaint employees in the automotive industry often raised in several informal discussions experienced by the author. Production managers complain about being accountable for difficult-to-reach production targets within very lean production timelines and that missing time might impact negatively on production. This was also previously reported on by George and Quinlan (2009) where the CEO of Lifeworks stated in an interview that the key factor affecting HCT participation was the extent of in-company support and/or sabotage, be it at management or at shop-floor levels. Negative perceptions of company support and confidentiality were also associated strongly with failure to participate in workplace HCT (Mundy & Dickinson 2004). Workers therefore cannot act on their workplace HCT intentions if external factors, for example, a lack of shift leaders' support, prevent them from doing so, making it essential for organisations to plan for an interruption in production time for HIV testing. It was important that in this study the interviewees explained how shop-floor workers felt reassured that they would be given the go-ahead to participate in HIV testing:

Ya, they [shift managers] will allow us throughout the day to go for testing.

Definitely, because, the way they organized this whole thing was very professional you know, each department had a chance, everybody had a chance you know, there wasn't the issue about no, busy building here, we're busy with production you won't be able to go. Stuff like that was out, you know. Everybody had a fair chance to go.

In addition, the prospect of lottery prizes helped change the way workers were thinking about the company. They viewed the introduction of the LIS as a sign that the company really cares about the workers rather than interpreting the initiative in a negative way:

 … ya, it [the lottery incentive system] made me think different because even the time they [shift managers] give us more time, they give us those dates that, that, there was a play that was played for the testing and all that stuff. So it made me think like, no, the company cares about us … 

Ah, to me I think I actually thought that this was a great move from management for the company as a whole you know. Implementing something like this … They [the company managers] also had a feeling or they had the knowledge that people you know, euh, face the stigma, myths and fears and that they wouldn't actually go for stuff like this – they thought that maybe we can make this creative and maybe pop in some prizes and stuff like that and maybe just maybe we could increase the numbers of people coming to test … .

It was a great idea, to put money in the whole programme.

It is likely that being given a fair chance to test and thus be entered into the LIS was further used as evidence that the company cares not only to allow them to test for HIV, but also wanted all to have an opportunity to enter into the LIS. Their appreciation for the concern the company showed in this instance seems to suggest that previous workplace health activities might not have been similarly supported especially when they were viewed as interfering with production and other company goals.

## 4. Conclusion

What emerges from the study is that the lottery incentives created excitement in the companies and increased an interest in workplace HCT. Lottery incentives were welcomed by the shop-floor workers and their partners and perceived as a supportive and innovative company approach to workplace HCT. The LIS facilitated open communication about HCT in the workplace and at home. The excitement created by the LIS facilitated social interactions that resulted in a strong group cohesion pertaining to HCT that mitigated the burden of HIV stigma in the workplace. In addition, the open discussions allowed for the development of supportive social group pressure to seek HCT as a collective in anticipation of a reward. This process was extended to the home where similar supportive communication with partners encouraged HCT behaviours.

The findings thus suggest that decision-making to participate in workplace HCT occurred at a social group level and that it was strongly influenced by normative considerations that were stimulated by the LIS at play. The LIS seemed to have been the impetus for collective processes in decision-making and behaviours and provided a supportive cultural context to facilitate HCT as a collective action. The findings demonstrate the importance of ‘collective’ support among shop-floor workers who share a more collective Afro-centric value system.

There are important implications for the designing of more effective workplace HCT interventions that encourage shop-floor workers to participate jointly in workplace HIV testing in South African manufacturing companies through the use of LIS. In particular, when designing and implementing LIS, one should also focus on cultural context and family rather than only on individual behaviours, as is commonly done in most HIV and AIDS interventions.

In the case of an LIS intervention it is important that the lottery's prizes, date of prize-giving and entry conditions are communicated sufficiently in advance to the HCT event to allow sufficient time for the communication process to occur around HIV testing and LIS to generate not only excitement but also social cohesion, which played a role in the uptake of workplace HCT in this study. Furthermore, peer educators, posters and leaflets also play an important role in reminding workers about the lottery incentive competition so as to increase excitement and anticipation. Finally, the HCT event should be organised as a short and intensive know-your-status event on the company's premises as it is important that many colleagues are seen to participate at the same time. Monitoring and evaluation of the LIS on shop-floor workers' experiences and HCT behaviours are important to improve understanding of the needs and concerns of employees and adapt interventions accordingly.

While the findings of this qualitative study cannot be generalised to all shop-floor workers of the participating companies or beyond, important insights into improving the uptake of HCT in workplaces have been provided. Questions remain however for further research and investigations. For example, would LIS have the desired effect on HCT behaviour in the context of other health screening options if lottery incentive entry was not dependent on HCT uptake?

The elucidation of how and why LIS encouraged workplace HCT showed the importance of providing an opportunity for shop-floor workers to participate jointly in workplace HCT to mitigate the burden of HIV stigma and discrimination. Furthermore, the significance of inter- and intra-player dialogue in activating supportive social norms for HIV testing in collectivist African contexts was highlighted.

## 5. Limitations

Due to the qualitative nature of the study the range of potential modifiers of responses to financial and other incentives including the type and magnitude of the incentive was not explored. However, the study explored employee experiences with a specific financial incentive system used in a specific company setting with a specific target group. Hence, the research does not necessarily allow for an understanding of the mechanisms of incentives for HCT among managers and administration staff. The study did not examine long-term effects of the LIS on shop-floor workers' HCT behaviour and could be best addressed in a quantitative, longitudinal study.
