# Corrigendum

**DOI:** 10.1080/17290376.2015.1020665

**Published:** 2015-03-04

Martin Weihs and Anna Meyer-Weitz (2014) A lottery incentive system to facilitate dialogue and social support for workplace HIV counselling and testing: a qualitative inquiry. *Journal of Social Aspects of HIV/AIDS*, 11(1).

http://dx.doi.org/10.1080/17290376.2014.937739

Author Martin Weihs would like to acknowledge a change in his affiliation between the date of first submission and acceptance of his article.

At the time of submission, the author was affiliated as ‘Technical Advisor to the Automotive Industry Development Centre Eastern Cape (AIDC EC) placed by the German Development Cooperation (GIZ), Port Elizabeth, South Africa’ as indicated by the published Version of Record.

Prior to acceptance of the article by the journal, the author's affiliation changed to ‘Senior Researcher, Human Sciences Research Council (HSRC), South Africa’. This should have been corrected in the article during the proofing stage of the production process.

The author would like to apologise for this oversight.

